# Clinician-centred interventions to increase vaginal birth after caesarean section (VBAC): a systematic review

**DOI:** 10.1186/s12884-015-0441-3

**Published:** 2015-02-05

**Authors:** Ingela Lundgren, Valerie Smith, Christina Nilsson, Katri Vehvilainen-Julkunen, Jane Nicoletti, Declan Devane, Annette Bernloehr, Evelien van Limbeek, Joan Lalor, Cecily Begley

**Affiliations:** Institute of Health and Care Sciences, The Sahlgrenska Academy at University of Gothenburg, Box 457, , SE-405 30 Gothenburg, Sweden; School of Nursing and Midwifery, Trinity College Dublin, 24 D’Olier Street, Dublin 2, Ireland; University of Eastern Finland, Faculty of Health Sciences, POB 1627, Kuopio University Hospital, 70211 Kuopio, Finland; Universita Degli Studi di Genova, Via Balbi 5, 16126 Genoa, Italy; School of Nursing and Midwifery, NUI Galway/West North West Hospital Group, University Road, Galway, Ireland; Department of Obstetrics, Gynaecology and Reproductive Medicine, Midwifery Research and Education Unit, Hannover Medical School, Carl-Neuberg-Straße 1, 30625 Hannover, Germany; Department of Midwifery Science, Zuyd University, POB 1256, 6201 BG Maastricht, The Netherlands

**Keywords:** VBAC, Systematic review, Interventions, Clinicians

## Abstract

**Background:**

The number of caesarean sections (CS) is increasing globally, and repeat CS after a previous CS is a significant contributor to the overall CS rate. Vaginal birth after caesarean (VBAC) can be seen as a real and viable option for most women with previous CS. To achieve success, however, women need the support of their clinicians (obstetricians and midwives). The aim of this study was to evaluate clinician-centred interventions designed to increase the rate of VBAC.

**Methods:**

The bibliographic databases of The Cochrane Library, PubMed, PsychINFO and CINAHL were searched for randomised controlled trials, including cluster randomised trials that evaluated the effectiveness of any intervention targeted directly at clinicians aimed at increasing VBAC rates. Included studies were appraised independently by two reviewers. Data were extracted independently by three reviewers. The quality of the included studies was assessed using the quality assessment tool, ‘Effective Public Health Practice Project’. The primary outcome measure was VBAC rates.

**Results:**

238 citations were screened, 255 were excluded by title and abstract. 11 full-text papers were reviewed; eight were excluded, resulting in three included papers. One study evaluated the effectiveness of antepartum x-ray pelvimetry (XRP) in 306 women with one previous CS. One study evaluated the effects of external peer review on CS birth in 45 hospitals, and the third evaluated opinion leader education and audit and feedback in 16 hospitals. The use of external peer review, audit and feedback had no significant effect on VBAC rates. An educational strategy delivered by an opinion leader significantly increased VBAC rates. The use of XRP significantly increased CS rates.

**Conclusions:**

This systematic review indicates that few studies have evaluated the effects of clinician-centred interventions on VBAC rates, and interventions are of varying types which limited the ability to meta-analyse data. A further limitation is that the included studies were performed during the late 1980s-1990s. An opinion leader educational strategy confers benefit for increasing VBAC rates. This strategy should be further studied in different maternity care settings and with professionals other than physicians only.

**Electronic supplementary material:**

The online version of this article (doi:10.1186/s12884-015-0441-3) contains supplementary material, which is available to authorized users.

## Background

Caesarean section (CS) rates have risen globally in the past decade, causing concern among clinicians. The lack of evidence of any decrease in morbidity associated with this rise ‘*raise questions about clinical effectiveness and the role of evidence* ([1], p. 78). Reasons suggested for the continuing increase in CSs include decreased training for clinicians in instrumental vaginal and vaginal breech births, medico-legal issues, the increased use of electronic fetal heart rate monitoring in labour [2-4], and maternal request [5,6]. Repeat CS after a previous CS birth is a significant contributor to overall increased CS rates and accounts for more than one-third of all CSs in the US [7].

Although a necessary and sometimes life-saving operation, CS is associated with more than double the rate of severe maternal morbidity and maternal mortality when compared with vaginal birth [8]. The challenge then is to reduce those CSs that are unnecessary, while retaining those that are needed to save lives and decrease morbidity.

Planned vaginal birth after CS (VBAC) compares favourably with routine elective repeat CS. A systematic review and meta-analysis of 203 studies [9], demonstrated that maternal mortality was increased significantly with elective repeat CS (ERCS) compared with planned VBAC (1.34 versus 0.38 per 10,000). In contrast, perinatal mortality was significantly increased with planned VBAC (13 per 10,000) compared with ERCS (5 per 10,000) although absolute rates are low [9]. This complicates the decision-making process as clinicians and women attempt to balance the risks involved. However, as maternal morbidity is also greatly increased with ERCS when compared to planned VBAC [9], the evidence, on balance, suggests that VBAC is a reasonable and safe option for most women. In Europe, VBAC rates vary widely, and have declined considerably in recent years, with significantly lower rates in Spain and Portugal (20-30%) than in Sweden, the Netherlands and Finland (45-55%) [10]. Although difficulties accessing tertiary care and medico-legal reasons may influence VBAC rates, variations are more likely to arise from an individual clinician’s approaches to decision-making around mode of birth [11]. Successful vaginal birth rates for women who plan a VBAC are high (70% to 87%) [10,12]; to achieve success, however, women need the support of their clinicians.

This systematic review was designed to identify, appraise and synthesise existing evidence that evaluated clinician-centred interventions designed to increase the rate of VBAC in women with a previous CS birth(s).

## Methods

### Criteria for selection of studies

Reports of randomised controlled trials including cluster randomised trials that evaluated the effectiveness of any clinician-centred intervention (defined as any intervention targeted directly at obstetricians and/or midwives or involving obstetricians and/or midwives as participants) designed to increase the rate of VBAC were considered eligible for inclusion in our review. Non-randomised studies and studies evaluating interventions to increase VBAC rates targeted at individuals other than clinicians were excluded. The primary outcome measure was VBAC, and the secondary outcomes were: compliance with intervention, modes of birth (instrumental birth, emergency CS, elective CS), maternal death, perinatal death and uterine rupture.

### Search strategy

We searched the electronic databases of The Cochrane Library (CENTRAL), PubMed, PsychINFO and CINAHL from their inception date to July 2014. The following search strategy was developed and adapted as appropriate to the various databases (Additional file 1).

The search string was reviewed for completeness and accuracy, using the peer review of electronic search strategies (PRESS) criteria [13], by a review team member not involved in the strategy development (CB). The PRESS criteria, developed through systematic review and expert opinion, facilitates independent review of the developed search strategy, prior to application, to enhance the quality of the search methodology in systematic reviews. Eleven criteria are listed which are used to guide the peer reviewer in assessing the developed search strategy; for example, assessment of whether the elements addressing the search question have been correctly combined with Boolean and/or proximity operators and assessment as to whether all relevant spelling variants are covered by the search terms. In addition, the selection of papers for inclusion in the review was performed independently by two teams of reviewers (IL and CN, and CB and JL). The web-based systematic review software DistillerSR (http://systematic-review.net/) was used to manage the search and citation screening process. DistillerSR is a 100% web-based package which allows reviewers download database identified citations. Independent screening by reviewers can then be performed from anywhere in real time using any web browser or type of computer. The system is designed to allow for identification of agreements by reviewers on inclusion and exclusion and movement of citations to next level screening (for example, where both reviewers agree on inclusion at abstract screening, the citation is forwarded for full text screening). In addition, any disagreements on inclusion at each level are highlighted for the reviewers. The package allows for rapid, easy and precise screening of papers for including in a systematic review.

### Quality assessment of included studies

The quality of the included studies was assessed using the quality assessment tool, ‘Effective Public Health Practice Project’ [14], which assists in assessing randomised trials for potential sources of bias. This tool assesses components such as bias in selection, allocation, blinding, confounding, methods used for data collection, withdrawals from the study, analysis and intervention integrity. Following assessment, each study is assigned a rating of Strong quality (no weak ratings noted), Moderate (one weak rating noted) or Weak (two or more weak ratings noted). If an individual study received a ‘Weak’ global rating score, due to poor methodological quality, this study was subsequently excluded from analysis.

Two members of the review team (VS and JN) assessed the quality of included studies independently. Any disagreements were discussed and resolved by consensus. Where disagreements occurred that could not be resolved by consensus, we planned to consult a third reviewer; however, this was not necessary.

### Data extraction and analysis

Three review team members (KVJ, AB and EvL) independently extracted data on outcomes of interest using a pre-designed data extraction form. The data were subsequently examined by a third reviewer (VS) for accuracy. We planned to perform meta-analyses of dichotomous data, and to use a summary risk ratio with 95% confidence intervals to present the results and to pool continuous data using the mean, or standardised mean, difference with 95% confidence intervals. Due to the diverse nature of the interventions that were evaluated in the individual studies, statistical pooling of individual study results was not possible. Consequently, we have provided a narrative synthesis of the results.

## Results

**Figure 1:**
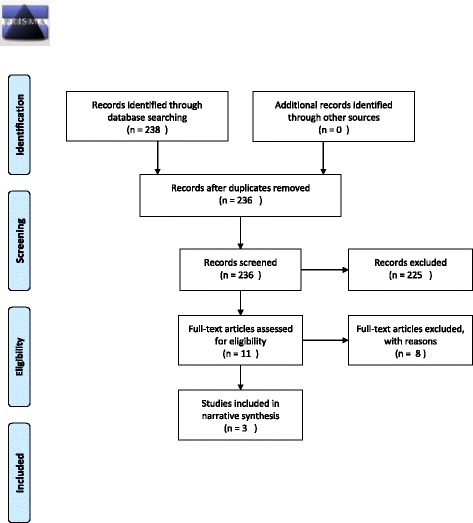
**Flow-diagram of the selection and search process.**

We identified 238 citations from the database search. After removing duplicates, 236 unique citations were screened by title and abstract and 225 of these were excluded. Full-text papers of the remaining eleven citations were obtained and reviewed. Eight of these were excluded: one did not focus on clinicians, two did not refer to a specific intervention, three did not focus on VBAC, one was a trial protocol and one was a review of trials of planned elective repeat caesarean section versus planned vaginal birth for women with a previous caesarean section (See List of Saints). This resulted in three papers suitable for inclusion in this review (Figure 1). One study [15] evaluated the effectiveness of antepartum x-ray pelvimetry (XRP) in women with one previous CS. One study [16] evaluated the effects of external peer review on CS birth and one study [17] evaluated opinion leader education and audit and feedback as methods for encouraging compliance with a guideline for the management of women with a previous CS.

### Excluded studies

Mancuso A, De Vivo A, Fanara G, Albiero A, Priolo AM, Giacobbe A, Franchi M. Caesarean section on request: Are there loco-regional factors influencing maternal choice? An Italian experience. *Journal of Obstetrics and Gynaecology,* 2008 28(4), 382–385.

Caroline A. Crowther CA, Dodd JM, Hiller JE, Haslam RR, Robinson JS, on behalf of the Birth After Caesarean Study Group. Planned Vaginal Birth or Elective Repeat Caesarean: Patient Preference Restricted Cohort with Nested Randomised Trial *PLoS Medicine* 2013, 9(3), e1001192.

Bernitz S, Aas E, Øian P. Economic evaluation of birth care in low-risk women. A comparison between a midwife-led birth unit and a standard obstetric unit within the same hospital in Norway. A randomised controlled trial. *Midwifery* 2013, 28, 591–599.

Lavender T, Kingdon C, Hart A, Gyte G, Gabbay M, Neilson JP. Could a randomised trial answer the controversy relating to elective caesarean section? National survey of consultant obstetricians and heads of midwifery. *BMJ* 2005, 331, 490–91.

Montgomery AM, Emmett CL, trial coordinator, Fahey T, Jones C, Ricketts I, Patel RR, Peters TJ, Murphy DJ, professor of obstetrics, on behalf of the DiAMOND Study Group. Two decision aids for mode of delivery among women with previous caesarean section: randomised controlled trial. *BMJ* 2007 4 doi:10.1136/bmj.39217.67101955.

Dodd JM, Crowther CA, Huertas E, Guise JM, Horey D. Planned elective repeat caesarean section versus planned vaginal birth for women with a previous caesarean birth. *Cochrane Database of Systematic Reviews* 2013, 12, CD004224.

Giguère A, Légaré F, Grimshaw J, Turcotte S, Fiander M, Grudniewicz A, Makosso-Kallyth S, Wolf FM, Farmer AP, Gagnon MP. Printed educational materials: effects on professional practice and healthcare outcomes. *Cochrane Database of Systematic Reviews* 2012, 10, CD004398.

Homer CSE, Besley K, Bell J, Davis D, Adams J, Porteous A, Foureur M. BMC Pregnancy and Childbirth 2013, 13:140. Does continuity of care impact decision making in the next birth after a caesarean section (VBAC)? A randomised controlled trial [http://www.biomedcentral.com/1471-2393/13/140].

### Description of included studies

#### X-ray Pelvimetry as an assessment for suitability for VBAC

Thubisi et al. [15] compared antepartum XRP with no antepartum XRP in women at 36 weeks gestation to determine mode of birth. Participants were pregnant women (n = 306) with one previous transverse lower segment CS and no obvious medical or obstetric risk factor (e.g. abnormal fetal lie, intrauterine death, intrauterine growth restriction, multiple pregnancy and maternal medical disorders, such as cardiac disease, contra-indicating a planned VBAC). One hundred and fifty-three women were initially randomised to each group, however, 18 women were subsequently excluded for the following reasons; four women withdrew (one and three in the intervention and control group, respectively) and 14 women were excluded due to pregnancy complications (eight in the intervention group and six in the control). Women in the intervention group received antenatal XRP at 36 weeks. If XRP measurements were above minimum values (i.e., saggital inlet 11 cm, saggital outlet 10 cm, transverse inlet 11.5 cm and transverse outlet 9 cm) women were permitted to attempt a planned VBAC (84 women). Women in the control group did not receive antenatal XRP but had a postpartum XRP (144 women). The main outcome measures of interest were mode of birth, including VBAC, maternal and perinatal morbidity and mortality and effect of antepartum XRP on the rate of repeat CS.

### Opinion leaders and audit and feedback of CS

Lomas et al. [17] evaluated two interventions in their study; audit and feed-back and use of opinion leaders. The unit of randomisation was a community hospital with at least 100 beds, of which 10 or more were obstetrical, that had no status as a teaching institution. Of 51 hospitals in 24 counties that satisfied the inclusion criteria, 16 hospitals were randomly selected to take part in one of the intervention groups or in the control group. Audit and feedback, which comprised of the following minimum activities; i) to establish departmentally agreed-on criteria for the use of CS in cases of women with previous CS, based on (but not identical to) the practice guideline, ii) to have medical audits of the charts of all women with a previous CS and to compare actual practice with the agreed-on criteria and iii) to hold meetings of the entire department every three months during 1988 for feedback and discussion of the audit results, especially discrepancies between actual criteria and agreed-on practice. Opinion leaders, which comprised of the following minimum activities: i) a mailing (early 1988) under the opinion leader’s name with a covering letter of an information binder for each physician engaged in obstetrical care in the opinion leader’s hospital (including the guideline in excerpt and in full version with a visual aid, a bibliography of relevant studies and letters of support for the guideline and the study); ii) a mailing (for later inclusion in the binder) of two further detailing sheets over the first months of 1988, addressing topics that the opinion leaders agreed were of concern to colleagues who might wish to consider implementing the recommendations of the practice guideline; iii) the opinion leader was to host a meeting with an expert speaker who was both knowledgeable and credible in the area of VBAC; and iv) to maintain and enhance their regular formal and informal educational contacts with colleagues and to record these in logbooks for the first 12 months of the intervention. The control group included all physicians active in obstetrical care who received a single mailing (January 1988) of a copy of the practice guideline. A brief exhortatory letter drew attention to the portion of the guideline that addressed the use of CS for women with a previous CS. The letter emphasised that the guideline had been endorsed by the national obstetrical specialty society, and requested that physicians implemented the recommendations. The total number of participants eligible for planned VBAC was 2496 (n = 524 in audit and feedback intervention, 739 in opinion leader intervention and 1233 in control group). The primary outcomes were rates of trial of labour and VBAC over the 24-month study period.

#### Peer-review of CS

Bickell et al. [16] evaluated the effectiveness of peer review on CS rates. External peer review was performed by ACOG-trained teams of three or four physicians and nurse reviewers who visited intervention hospitals (n = 45), interviewed key staff members, and reviewed labour and birth records to assess the quality of care. Records were selected randomly using the New York State Department of Health hospital discharge data base. Review teams provided feedback to the hospital through an exit interview, written summary of findings and recommendations. Outcomes (rates of CS and VBAC) were compared to non-peer reviewed hospitals (n = 120) for the years before and after completion of the programme (1988–1993).

### Methodological quality of included studies

Due to the nature of the intervention, blinding of either the clinician or the participating woman to her allocation was not feasible. Therefore, a lack of blinding did not negatively affect the quality assessment (Table 1).

**Table 1:** **Results of the methodological quality appraisal (Effective Public Health Practice Project) of the included studies**

**Component**	**Thubisi**	**Bickell**	**Lomas**
Selection bias avoided	Strong	Strong	Strong
Allocation bias avoided	Moderate	Strong	Moderate
Confounders avoided	Strong	Strong	Strong
Blinding (but not considered when calculating global quality score)	Weak	Weak	Weak
Data collection methods	Weak	Strong	Strong
Withdrawals & Drop-outs	Strong	Strong	Strong
Analysis: Intention to treat	Strong	Strong	Weak
Intervention integrity: % of participants that received allocated intervention	Strong	Strong	Strong
**Global quality score**	**Moderate**	**Strong**	**Moderate**
**Include study**	**Yes**	**Yes**	**Yes**

### Effects of interventions

We did not regard the three interventions studied to be sufficiently similar to ensure meaningful conclusions from a statistically pooled result. Therefore, a narrative synthesis of results is reported by presenting the major outcomes and results, organised by intervention categories. Forest plots illustrating point estimates (relative risks, (RR) and 95% confidence intervals (95% CIs) for each study for each of the main outcomes are presented. All of the studies reported the primary outcome of VBAC rates. Data on our pre-specified secondary outcomes were limited or reported variously in the included studies. Consequently, the primary outcome of interest only is reported in this review.

### Antenatal XRP versus no antenatal XRP

**Figure 2:**
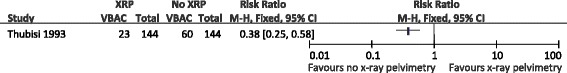
**VBAC – antepartum X-ray pelvimetry (XRP) versus no antepartum X-ray pelvimetry.**

Thubisi et al. [15] evaluated antenatal XRP in women with one previous CS. Women receiving antenatal XRP were statistically significantly less likely to have a VBAC than women who did not undergo XRP in pregnancy (16% versus 42%) (Figure 2). When women in the intervention group, with an inadequate antenatal XRP (as per study protocol), were excluded from the analysis (60 women), the results remained statistically significant in favour of no antenatal XRP for increasing VBAC rates (27% versus 42%; RR 0.66, 95% CI 0.44-0.98).

### Opinion leaders, audit and feedback of CS

**Figure 3:**
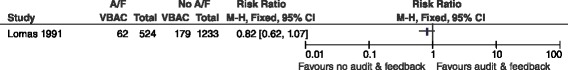
**VBAC – audit and feed-back (A/F) versus no audit and feed-back.**

**Figure 4:**
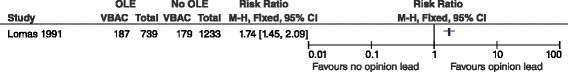
**VBAC – opinion leaders (OLE) versus no opinion leaders.**

In Lomas et al’s [17] study, the analysis demonstrated no statistically significant difference in the incidence of VBAC between the audit and feedback (A/F) and control groups (12% versus 14%, respectively) (Figure 3). In contrast, an opinion leader education strategy (OLE) significantly increased the VBAC rate (25%) when compared to the control group (14%) (Figure 4).

### Peer review of CS

**Figure 5:**
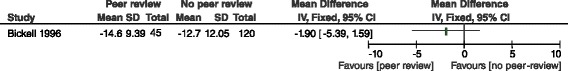
**VBAC – peer review versus no peer review.**

Bickell et al. [16] reported the mean proportion of VBACs across all participating hospitals for the years 1988 (the year peer review was introduced) to 1993. The proportion of VBACs increased in both peer reviewed and non-peer reviewed hospitals during the study period (by 14.6% and 12.7%, respectively). The increase in mean VBAC proportions between peer-reviewed and non-peer-reviewed hospitals was not statistically significant (Figure 5).

## Discussion

### Main findings

This systematic review of clinician-centred interventions for increasing VBAC rates demonstrates that the use of opinion leaders significantly increases the rates of VBAC where the use of antenatal XRP significantly decreases VBAC rates. External peer review of CS did not demonstrate any statistically significant effect in either direction.

### Strengths and limitations

This was a comprehensive review, covering all key health-related databases from their inception. We found relatively few studies evaluating the effects of clinician-centred interventions on VBAC rates and, of those we did find, the interventions evaluated are of varying types. Of the three included studies, the methodological quality was judged to be either moderate or strong, adding strength to the findings of this review. All three included studies scored strongly on ‘selection bias avoided’ as the individuals selected to participate in the study were very likely to be representative of the target population. Thubisi received an overall ‘moderate’ score for allocation bias as alternative randomisation was used to ensure equal groups. A weak rating was allocated to the data collection component for this study as we were unable to determine from the paper whether the data collection tools/methods were reliable and valid. All three studies reported clearly their withdrawal/drop-out rates, receiving a component rating of strong on this methodological criterion. Finally, although a number of women (n = 60) in the Thubisi study did not receive the allocated XRP intervention due to strict protocol criteria, to receive a strong rating on this component the overall proportion of participants not receiving their allocated intervention must be greater than or equal to 80%. When the numbers in both intervention and control groups were considered, 79.7% overall received their assigned allocation. For this reason, we allocated a strong quality rating for this component in the Thubisi study. For the other two studies, greater than 80% of the participants received their assigned allocation and were thus deemed methodologically strong on this component. One limitation of this review is that the included studies are rather old. Since the studies were performed the CS rate has increased in the included countries and the maternity care may also have changed. We had presumed to find more, and recently performed, studies.

### Interpretation

The study by Lomas et al. [17], with 76 physicians from 16 community hospitals in Canada, demonstrated that an educational opinion leader strategy significantly increases the VBAC rate when compared to a control (i.e. a single mailing of a copy of the practice guideline with a brief exhortatory letter drawing attention to the portion of the guideline that addressed the use of CS for women with a previous CS, and request for physicians to implement the recommendations). The educational opinion leaders supported their colleagues [17], which highlights the importance of learning from professional experts and following-up initial educational endeavors. According to this study, common guidelines are insufficient and should be combined with an educational strategy facilitated by an opinion-leader. These findings are supported by a review by Khunpradit et al. that examines non-clinical interventions, applied independently of patient care in a clinical encounter, for reducing unnecessary CS [18]. In this review, guidelines with support of local opinion leaders, internal peer review and mandatory second opinion were shown to be effective in reducing CS rates [19]. Although the study by Lomas et al. [17] was conducted in the late 1980s, we judge that the findings are of relevance today. The study assessed behavior change strategies and innovative education that improved the quality of care. These complex questions remain as important and relevant today as they did then. Further research, in the form of methodologically robust randomized trials, are needed to evaluate this strategy in different maternity care systems and countries. As concluded in a population-based cohort study about the rising CS rate in Australia, only 24% of the increase in primary CS rates could be explained by maternal factors and by increased private maternity services, suggesting that changing attitudes towards CS birth are, in part, driving the increase [19].

The study by Thubisi et al. [15] does not support the hypothesis that routine antepartum XRP in women with a history of a previous CS effectively identifies those who can achieve a vaginal birth. Rather, the authors conclude that more women will succeed in giving birth vaginally without any additional harm to themselves and their babies if antepartum XRP is not performed. This conclusion is supported by recent guidelines recommending that the use of X-ray pelvimetry to decide about planned VBAC is associated with an increase in the repeat CS rate without any reduction in the rate of uterine rupture [20]. Sun and Wen [21] refer to the study by Thubisi et al. [15] as one of few randomised trials that studied the clinical usefulness of X-ray pelvimetry. Therefore the study is relevant today even if it was performed more than 20 years ago. The authors [21] conclude that it would be better that x-ray pelvimetry is performed as a complementary treatment in women for whom a trial of labour after CS is planned, rather than performed as routine.

Since the rising CS rate over time is of international concern [1], we anticipated that we would have found more data on this phenomenon. Lomas et al. concluded that there were no adverse clinical outcomes attributed to the interventions, and the use of an opinion leader improved the quality of care. However, 74.2% of the women were offered VBAC and 38.2% experienced a successful VBAC which, according to the authors, may be due to women’s expressed preferences, or ‘patient factors’. Further advances may therefore have to rely on the education of women [17] on the advantages of planned VBAC when compared with repeat planned CS. We support that conclusion but would also suggest that other health professionals involved in VBAC-care, in addition to obstetricians, should be included. A study from Italy exploring professionals’ (midwives and physicians) attitudes demonstrated differing attitudes towards CS according to professional roles [22]. Midwives appeared to be more aware of the risk of performing unnecessary CS, whereas obstetricians were more likely to underestimate risk of CS and to overestimate the benefits of this procedure. The authors conclude that midwives discussing the risks and benefits of CS with women before birth could have a positive influence on VBAC rates. We suggest that further studies should include both midwifery and obstetric opinion leaders in order to validate this as an effective intervention to increase VBAC rates internationally.

Since the Lomas study [17] the CS rate has increased in Canada from 16.4% in 1995 to 23.3% in 2006, which occurred with no change in perinatal mortality [23]. The VBAC rate in Quebec has declined from nearly 40% in 1995 to 20% in 2009. Rossignol et al. [23] obtained statistical variations of CS rates over time, across Canadian regions, and within professional practices from 1969–2009. The results show that expectant management (as an alternative to labour induction) and planned vaginal birth after CS is the leading robust strategy to reduce rates of CS in women at low risk of obstetric complications. According to the authors, increasing the availability of VBAC would require appropriate identification of potential candidates (currently still a barrier), as well as specially trained professionals in centers that can ensure safety [23]. The authors conclude that the major argument against reducing the rate of CS remains the fear of legal action against clinicians for not intervening in the case of an adverse outcome. Fear of litigation is also supported by other authors as a major contributory factor to rising CS rates [17,23-25]. A study on obstetricians’ attitudes to CS in eight European countries (Luxembourg, the Netherlands, Sweden, France, Germany, Italy, Spain and UK) found that fear of litigation was less relevant to physicians’ decision-making in Sweden and the Netherlands, a finding consistent with the low medico-legal burden in these countries [24]. Sweden and the Netherlands have high VBAC rates of 45-55% [10], even higher than the peak rate in Canada in 1995 [24]. Therefore studies evaluating clinician-centred interventions for improving the VBAC rate must be related to a country’s culture and maternity care settings. According to Chandrahan and Arulkumaran [25] medico-legal problems in obstetrics can be reduced by effective communication, team working, training and education, a finding that supports involving more professionals than physicians in interventions for increasing the VBAC rate.

## Conclusions

The findings from this systematic review of clinician-centred interventions to increase VBAC identified only three studies that met the inclusion criteria, highlighting limited research in this area. The findings show that the use of opinion-leaders in women with previous CS increases the VBAC rate, and the use of antepartum XRP decreases the VBAC rate. There is a need for further research that evaluates interventions for increasing VBAC rates that target clinicians. In addition, an evaluation of the use of opinion-leaders in different maternity care settings and with professionals other than physicians is recommended.

## Additional file

Additional file 1:
**Search string.**
