# Supplementary material for: Clinician-centred interventions to increase vaginal birth after caesarean section (VBAC): a systematic review
Source: BMC Pregnancy Childbirth. 2015 Feb 5;15:16. doi: 10.1186/s12884-015-0441-3 (PMC4324420; doi:10.1186/s12884-015-0441-3)
Supplement: Additional file 1: — Search string. [file 12884_2015_441_MOESM1_ESM.pdf]

Additional file 1:

Search string

"Vaginal Birth after Cesarean"[Mesh] OR (("TOL" OR "Vaginal Birth" OR "normal birth" OR ventouse OR forcep* OR instrumental) AND (Cesar* OR Caesar* OR "c-section" OR "c-sections")) OR "VBAC" OR "TOLAC" AND "Health Personnel"[Mesh] OR "Professional-Patient Relations"[Mesh] OR "Professional-Family Relations"[Mesh] OR "Professional Practice"[Mesh] OR "Professional Autonomy"[Mesh] OR "Professional Role"[Mesh] OR "Delegation, Professional"[Mesh] OR "Education, Professional"[Mesh] OR "Education, Public Health Professional"[Mesh] OR "Physician's Practice Patterns"[Mesh] OR "Liability, Legal"[Mesh] OR "Malpractice"[Mesh] OR "Refusal to Treat"[Mesh] OR "Nursing, Team"[Mesh] OR "Standard of Care"[Mesh] OR "Personal Autonomy"[Mesh] OR "Clinical Nursing Research"[Mesh] OR "Nurse Midwives"[Mesh] OR "Medical Staff"[Mesh] OR "Medical Staff, Hospital"[Mesh] OR "Interprofessional Relations"[Mesh] OR "Decision Making, Computer-Assisted"[Mesh] OR "Consultants"[Mesh] OR "Practice Guideline" [Publication Type] OR "Midwifery"[Mesh] OR midwives OR midwife OR "obstetric nurse" OR "obstetric nurses" OR obstetrician* OR gynecologist* OR gynaecologist* OR physician* OR clinician* OR doctor OR doctors AND "Randomized Controlled Trial"[Publication Type] OR "Controlled Clinical Trial"[Publication Type] OR "Randomized Controlled Trials as Topic"[Mesh Terms] OR "Placebos"[Mesh Terms] OR (random* AND trial*[tiab]) OR "randomized"[tiab] OR "randomly"[tiab] or placebo*
